# Superior operational stability of immobilized l-asparaginase over surface-modified carbon nanotubes

**DOI:** 10.1038/s41598-021-00841-2

**Published:** 2021-11-02

**Authors:** Mafalda R. Almeida, Raquel O. Cristóvão, Maria A. Barros, João C. F. Nunes, Rui A. R. Boaventura, José M. Loureiro, Joaquim L. Faria, Márcia C. Neves, Mara G. Freire, Valéria C. Santos-Ebinuma, Ana P. M. Tavares, Cláudia G. Silva

**Affiliations:** 1 CICECO-Aveiro Institute of Materials, Department of Chemistry, University of Aveiro, 3810-193 Aveiro, Portugal; 2 Laboratory of Separation and Reaction Engineering - Laboratory of Catalysis and Materials (LSRE-LCM), Department of Chemical Engineering, Faculty of Engineering, University of Porto, Rua do Dr. Roberto Frias, 4200-465 Porto, Portugal; 3 Department of Engineering Bioprocess and Biotechnology, School of Pharmaceutical Sciences, São Paulo State University (Unesp), Araraquara, Brazil

**Keywords:** Immobilized enzymes, Immobilized enzymes, Biomaterials - proteins, Chemical engineering, Biocatalysis, Carbon nanotubes and fullerenes

## Abstract

l-asparaginase (ASNase, EC 3.5.1.1) is an enzyme that catalyzes the l-asparagine hydrolysis into l-aspartic acid and ammonia, being mainly applied in pharmaceutical and food industries. However, some disadvantages are associated with its free form, such as the ASNase short half-life, which may be overcome by enzyme immobilization. In this work, the immobilization of ASNase by adsorption over pristine and modified multi-walled carbon nanotubes (MWCNTs) was investigated, the latter corresponding to functionalized MWCNTs through a hydrothermal oxidation treatment. Different operating conditions, including pH, contact time and ASNase/MWCNT mass ratio, as well as the operational stability of the immobilized ASNase, were evaluated. For comparison purposes, data regarding the ASNase immobilization with pristine MWCNT was detailed. The characterization of the ASNase-MWCNT bioconjugate was addressed using different techniques, namely Transmission Electron Microscopy (TEM), Thermogravimetric Analysis (TGA) and Raman spectroscopy. Functionalized MWCNTs showed promising results, with an immobilization yield and a relative recovered activity of commercial ASNase above 95% under the optimized adsorption conditions (pH 8, 60 min of contact and 1.5 × 10^–3^ g mL^−1^ of ASNase). The ASNase-MWCNT bioconjugate also showed improved enzyme operational stability (6 consecutive reaction cycles without activity loss), paving the way for its use in industrial processes.

## Introduction

l-asparaginase (ASNase, EC 3.5.1.1) is an enzyme that catalyzes l-asparagine hydrolysis into l-aspartic acid and ammonia^1^, being present in different microorganisms such as bacteria, fungi, plant tissues, algae and in some animals^2^. This enzyme has important applications, mainly in the food and pharmaceutical industries. Specifically, in the food industry, ASNase prevents the formation of acrylamide (formed by the Maillard reaction that occurs at extreme temperatures between l-asparagine and carbonyl compounds), a neurotoxic, genotoxic, carcinogenic, and toxic compound^1^. On the other hand, in the pharmaceutical industry, ASNase is used as a biopharmaceutical to treat acute lymphoblastic leukemia and other malignant diseases, including Hodgkin’s disease and different types of leukemia or sarcoma^3^. The chemotherapeutic effect of ASNase is based on the catalysis of the l-asparagine present in blood, an essential amino acid for the growth of both normal and cancer cells^4^. Since cancer cells do not have l-asparagine synthetase, they must use the existing l-asparagine in the blood. Thus, ASNase achieves its antileukemic effect by depleting circulating asparagine and depriving cancer cells of asparagine^3^. Additionally, ASNase can be used in biosensor technology to detect asparagine levels in both sectors^3^. For instance, Li et al.^5^ produced an ammonium selective sensor based on adsorbed thermostable and recombinant ASNase biosensor from *Archaeoglobus fulgidus* cloned and expressed in *Escherichia coli*.

ASNase currently commercialized is produced from two primary bacterial sources, namely both recombinant *E. coli* and *Erwinia chrysanthemi*. Due to characteristics of its recombinant origin, its half-life and intrinsic activity decrease while its immunogenicity increases^2^. To overcome these disadvantages, Ulu and Ates^3^ recently reviewed natural and synthetic carriers (from erythrocytes to poly(ethylene glycol) (PEG)) and different techniques (chemical and physical methods) applied for ASNase immobilization. Immobilized enzymes have been widely used due to their improved stability and performance, and reusability^6^. However, the selection of the solid support and the procedure adopted for the enzyme immobilization significantly influences the resulting properties^6^.

Currently, nanomaterials and nanostructures have been widely applied as carriers for enzymes or proteins because of their high surface area and high enzyme/protein loading capacity^7^. Nanomaterials include several structures like nanoparticles, nanofibers and nanotubes, of which carbon nanotubes (CNTs) have been under extensive research in different fields, such as optics, electronics and catalysis^8^. CNTs are formed by a network of carbon atoms (graphene sheets) assembled into seamless cylinders of one (single-walled carbon nanotubes, SWCNTs, with a diameter of 0.4 to 3 nm)^9^ or more layers (multi-walled carbon nanotubes, MWCNTs, with diameters of about 5 to 100 nm) with either open or closed ends^10^. Additionally, MWCNTS can be classified according to two possible arrangements with implications on their properties: when the graphene sheets are arranged in nested concentric cylinders and when one graphene sheet is spirally wraparound on itself^11^. Moreover, CNTs' surfaces can be easily functionalized, tuning their properties towards specific applications and enhancing their efficiency either as supports or catalysts^12–14^.

CNTs have been reported as promising nanomaterials for diverse (bio)applications since relatively strong chemical interactions between biomolecules and CNTs are stablished, increasing the stability of the immobilized biomolecules towards harsh reaction conditions and enhance its reusability^15^. Specifically, the interaction of CNTs with biomolecules such as enzymes and proteins has been studied for laccase, peroxidase, organophosphate hydrolase, esterase, fibronectin, catalase, soy protein, among others^12,13,16–25^. The two main approaches for biomolecule attachment on CNTs are noncovalent and covalent immobilization. In the first one, the biomolecule is physically adsorbed on the CNT by hydrogen bonding or by hydrophobic, electrostatic, or π-π stacking interactions. In the second one, the biomolecule is covalently linked to the CNT by esterification or amidation of previously oxidized CNTs, by covalent attachment of the functional groups over the CNTs surface, or by linking molecules, which act as “bridges” between the CNTs and the biomolecule^26^.

Regarding CNTs biocompatibility envisioning the aforementioned applications, recent studies have been reporting the reduction of CNTs toxicity through their functionalization. For instance, Dong et al.^27^ showed that acid oxidation of CNTs leads to a decrease of CNTs cytotoxicity; their biocompatibility was improved when under direct incubation with human epithelial cells (BEAS-2B cells) and with enzymes (soybean peroxidase). Jain et al.^28^ evaluated the in vivo toxicity of pristine and acid-oxidized multi-walled carbon nanotubes. It was found that acidic MWCNTs are less toxic and more biocompatible than their pristine counterparts, and that the toxicity of MWCNTs depends on their functionalization density, i.e., the higher the density of surface carboxyl groups, the lower was the MWCNTs toxicity^28^. Moreover, quantitative biodistribution studies in mice showed a rapid elimination of highly oxidized MWCNTs from the organism, while pristine and less oxidized MWCNTs revealed greater retention time^28^.

In a previous work we have found that ASNase may be efficiently immobilized by a simple physical adsorption method over pristine carbon nanotubes^29^. Although we have obtained very promising results in terms of ASNase activity, the presence of stronger interactions between the carbon phase and the enzyme is highly desired from the technological point of view since they normally result in bioconjugates with higher reusability^29^. Surface functionalization of CNTs is generally applied for this purpose.

To the best of our knowledge, up to now only two works have been published on the immobilization of ASNase over functionalized CNTs^30,31^. Ulu et al.^30^ reported the use of calcium-alginate/MWCNTs hybrid beads for the immobilization *E. coli* ASNase while Haroun et al.^31^ described the immobilization of partial purified *Aspergillus versicolor* ASNase onto oxidized MWCNTs.

In the present work, MWCNTs functionalized through a simple hydrothermal oxidation route with nitric acid were used for the immobilization of commercial *E. coli* ASNase and the results compared with those obtained with the unmodified material^29^, and by Haroun et al.^31^, which used similar materials. Although, we propose a comparable strategy to the one developed applied by Haroun et al.^31^, the immobilization process developed by us used an ASNase from a different microorganism (Haroun et al.^31^ immobilized ASNase produced by a fungi, *A. versicolor*, while we immobilized ASNase produced by a bacteria, *E. coli*). Although, both microorganisms are good sources of ASNase, the fungal complex morphology can be critical for the feasibility of scaling up the process since fungal cultivation in bioreactor is sensible to several parameters, such as oxygen supply and transfer, inoculum size, pH, and stirring. Both enzymes have a base of similarity, but present significant differences in what concerns their structure and physicochemical properties^1^. In this sense, the immobilization behavior can be significantly different and for this reason we developed the present work to expand the characterization of *E. coli* ASNase immobilization. Different operation conditions, including pH, contact time and ASNase/MWCNT mass ratio were evaluated. The immobilization of the enzyme was evidenced by characterization of the ASNase-MWCNT bioconjugate using different techniques. Moreover, the operational stability was assessed by determining the efficiency of the ASNase/MWCNT over a series of consecutive cycles.

## Results and discussion

### ASNase immobilization on functionalized MWCNTs

The effect of several parameters, namely pH, contact time and ASNase/MWCNT mass ratio, on the enzyme immobilization yield and enzymatic activity was evaluated. The ASNase-MWCNTs bioconjugates were prepared using MWCNTs functionalized with different HNO_3_ concentrations, and for comparison purposes, pristine MWCNT was also tested.

#### Effect of the pH on ASNase immobilization

**Figure 1:**
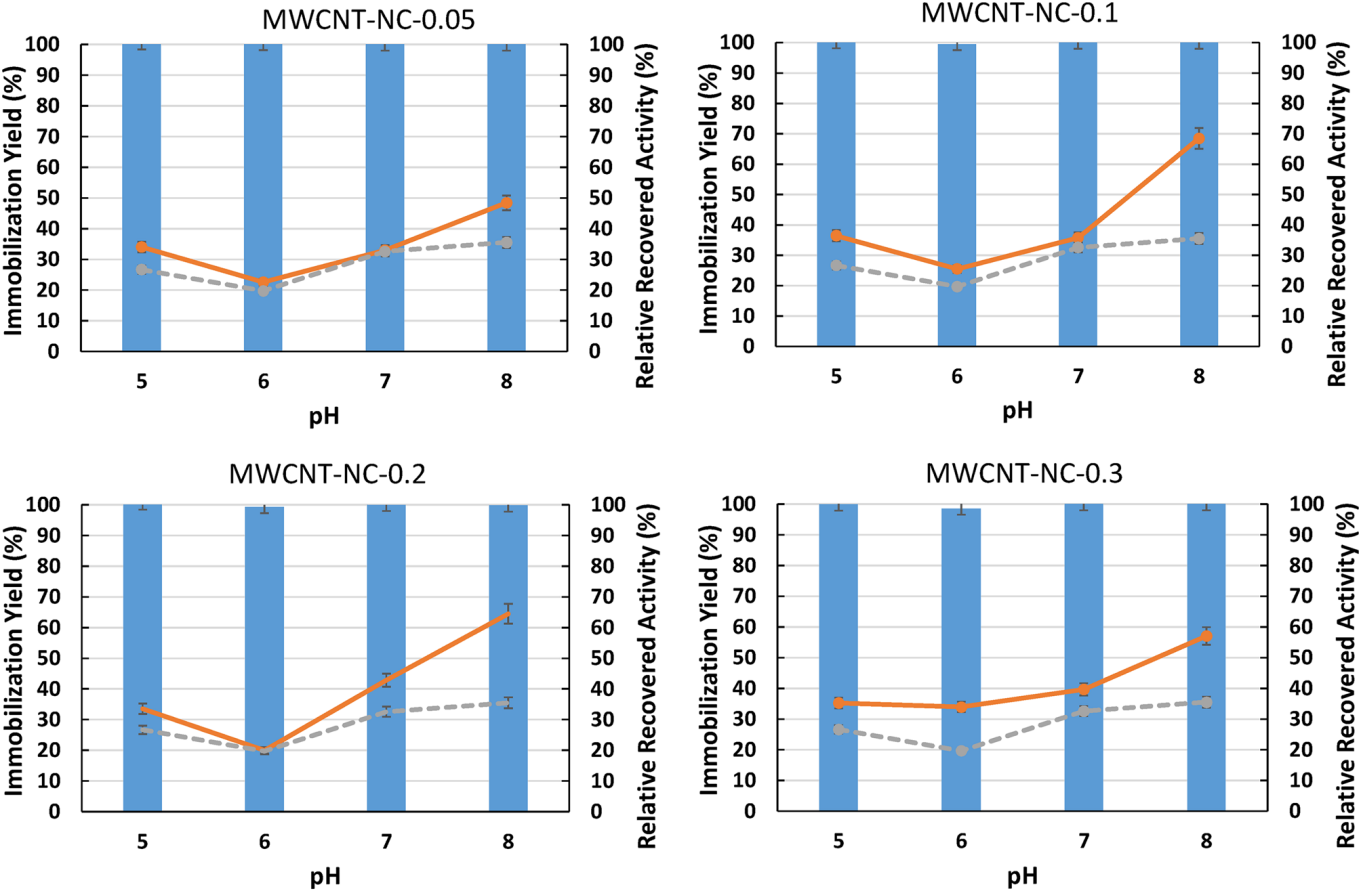
Effect of pH on immobilization yield (columns) and relative recovered activity (orange lines) obtained with the immobilization of 8.6 × 10^–5^ g mL^−1^ of ASNase on 2 mg of functionalized MWCNTs (functionalization with HNO_3_ aqueous solutions with variable concentrations: 0.05, 0.10, 0.20 and 0.30 M) for 60 min of contact time. Grey lines represent the relative recovered activity of ASNase on 2 mg of MWCNTs without any functionalization (MWCNT-NC, control).

In order to evaluate possible electrostatic interactions between ASNase and MWCNTs, firstly, the pH effect was evaluated at distinct pH values ranging from 5.0 to 8.0, whilst keeping the ASNase concentration at 8.6 × 10^–5^ g mL^−1^ (1.72 × 10^–5^ g of ASNase) and contact time of 60 min. To this end, the isoelectric point of ASNase was determined, obtaining a value of 5.2 (Figure S1 in the Supporting Information). This value follows the literature, where isoelectric points of ASNase ranging from 5.0 to 5.7 have been reported^32^. The results of ASNase immobilization yield and relative recovered activity are depicted in Fig. 1 (detailed data in Table S1 in the Supporting Information). According to Fig. 1, practically total enzyme adsorption was achieved in the pH range under study, with an immobilization yield above 98% (more than 1.62 × 10^–5^ g of ASNase was immobilized) for all functionalized MWCNTs.

Under the studied conditions, the enzyme is partially positively charged in the assays performed at pH 5.0; at pH 6 and above, the enzyme is negatively charged. Additionally, as the point of zero charge of the modified MWCNTs used is around 3.0, the nanomaterials are negatively charged in all the assays performed^22^, which agrees with the metallic-like conductive properties of these materials. Thus, since in most pH values addressed the enzyme and MWCNTs are negatively charged, a repulsion between them would expected if electrostatic interactions are playing a role. Furthermore, no significant differences exist in the immobilization yields obtained at and above pH 5. Therefore, electrostatic interactions between the material and the enzyme do not play a major role, with hydrophobic (hydrophobic regions on enzyme exterior can interact with the wall of CNTs through hydrophobic interaction) and $\pi$–$\pi$ stacking interactions (interaction between the sidewalls of CNTs and the ASNase aromatic rings) appearing as the major forces in the adsorption of ASNase^33^.

High ASNase relative recovered activities (RRA) were detected at pH 8.0, and low RRA at pH 6.0, meaning that the pH influences the activity of ASNase after immobilization. Probably, at low pH values (below 7.5), the active site of the immobilized enzyme is partially deactivated since it is known that ASNase is sensitive to high H^+^ concentrations^34,35^. Regarding the effect of the MWCNTs functionalization on the ASNase RRA, it is evident that a higher RRA was obtained when functionalized MWCNTs are used (Fig. 1—orange lines) over non-functionalized ones (Fig. 1—grey lines). Moreover, a similar tendency of the RRA increase with the increase in pH is observed for all degrees of MWCNTs surface functionalization. A maximum RRA of 69% was attained with the functionalized MWCNT-NC-0.1, followed by MWCNT-NC-0.2, MWCNT-NC-0.3 and MWCNT-NC-0.05. A detailed discussion about the effect of surface modification is presented below, together with the effect of contact time.

Considering high ASNase RRA values were detected at pH 8.0, this pH was selected for further assays.

#### Contact time effect on ASNase immobilization

**Figure 2:**
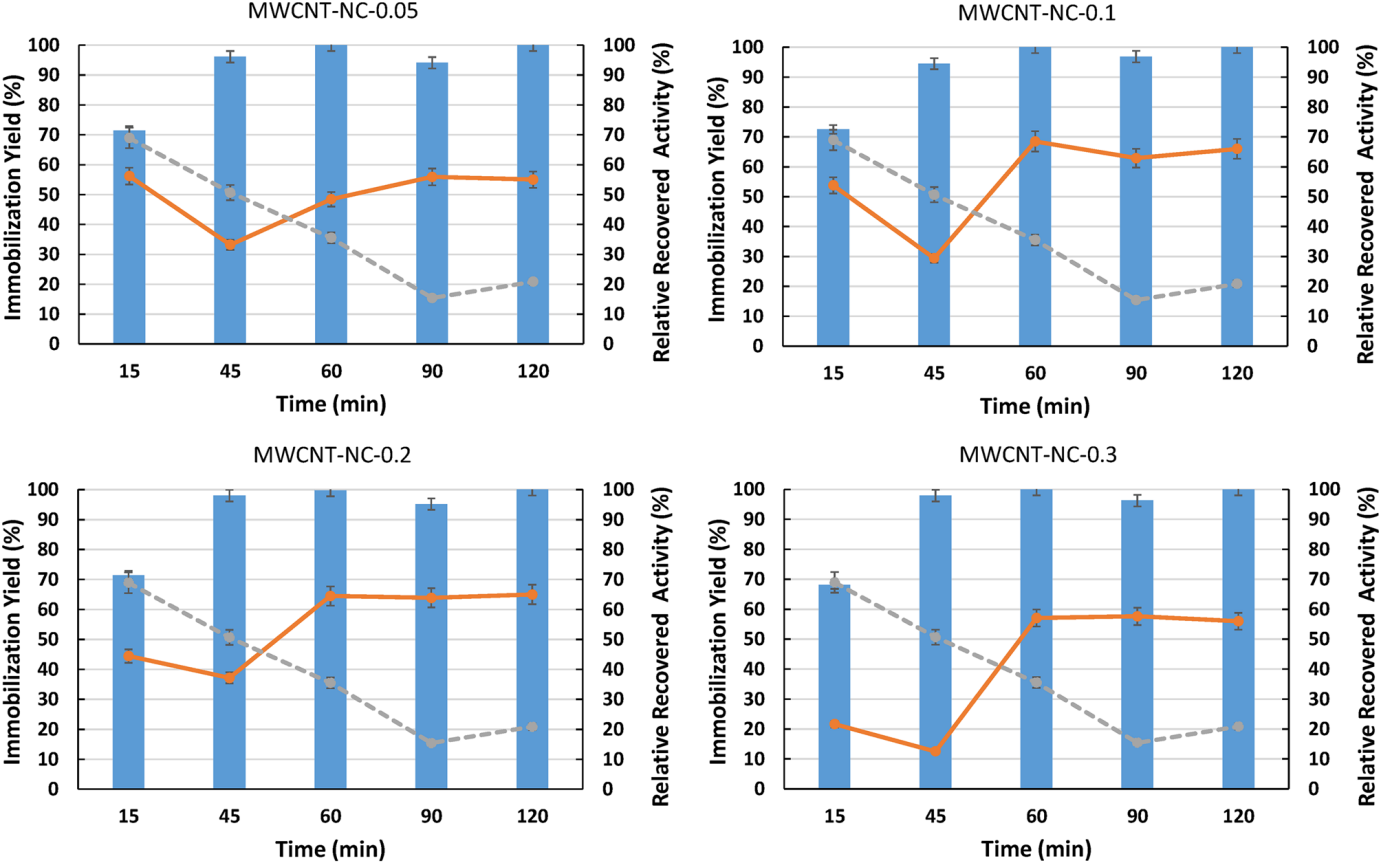
Effect of contact time on immobilization yield (columns) and relative recovered activity (orange lines) obtained with the immobilization of 8.6 × 10^–5^ g mL^−1^ of ASNase on 2 mg of functionalized MWCNTs (functionalization with HNO_3_ aqueous solutions with variable concentrations: 0.05, 0.10, 0.20 and 0.30 M) at pH 8. Grey lines represent the relative recovered activity of ASNase on 2 mg of MWCNTs without any functionalization (MWCNT-NC, control).

The influence of contact time on the ASNase immobilization was also addressed using the selected pH of 8 and all modified MWCNTs. Five contact times, between 15 and 120 min were evaluated. The results presented in Fig. 2 (detailed data in Table S2 in the Supporting Information) reveal that contact time significantly affects the ASNase immobilization yield and RRA. The contact time of 15 min promotes the lowest values of immobilization yield ($\sim$ 70%, $\sim$ 1.20 × 10^–5^ g of ASNase was immobilized) for all MWCNTS used. These results may be due to the short period of contact between the enzyme and the MWCNTS, not allowing the complete attachment of ASNase. On the other hand, after 60 min of contact time, the highest values of immobilization yield were reached (higher than 90%, more than 1.55 × 10^–5^ g of ASNase was immobilized) for all the MWCNTS.

Regarding the RRA, the same behavior was observed, 15 min of contact time was not enough to obtain high ASNase RRA values for all MWCNTs studied (12–37%). However, after 60 min, maximum ASNase RRA values were attained (58–69%), except for MWCNT-NC-0.05, which needs 90 min to attain 56% of RRA, probably due to a lower extent of surface functionalization. Among the functionalized MWCNTs, the best results were obtained with MWCNT-NC-0.1 (as previously reported for the assays regarding the pH effect). Comparing to non-functionalized MWCNTs, the previous results with surface-modified MWCNTs exhibit an opposite behavior (Fig. 2-grey lines).

It is clear that the physical and chemical properties of the MWCNTs influence the catalytic performance of ASNase. The RRA of ASNase immobilized in pristine MWCNTs decreased with the increase in contact time, suggesting that the oxidative modification of MWCNTs has a beneficial protector effect on the ASNase RRA. It is recognized that the acid oxidation treatment modifies the physical and chemical properties of CNTs, leading to an improvement of CNTs biocompatibility^27^. In the present work, when MWCNTs are oxidized, their hydrophilicity increases due to the introduction of oxygen-containing functional groups, i.e., carboxyl, carbonyl, and phenol groups, as described below. This surface modification may promote a proper interaction between the enzyme and the nanomaterial, thus leading to an increase in the RRA of ASNase^36^. Besides, metal catalyst particles present in the surface of MWCNTs, which can inhibit the ASNase activity, are removed during the acidic treatment^37^. Considering immobilization yields of 100% and RRA values higher than 50%, 60 min of contact time was chosen for further studies.

#### Enzyme concentration effect on ASNase immobilization

**Figure 3:**
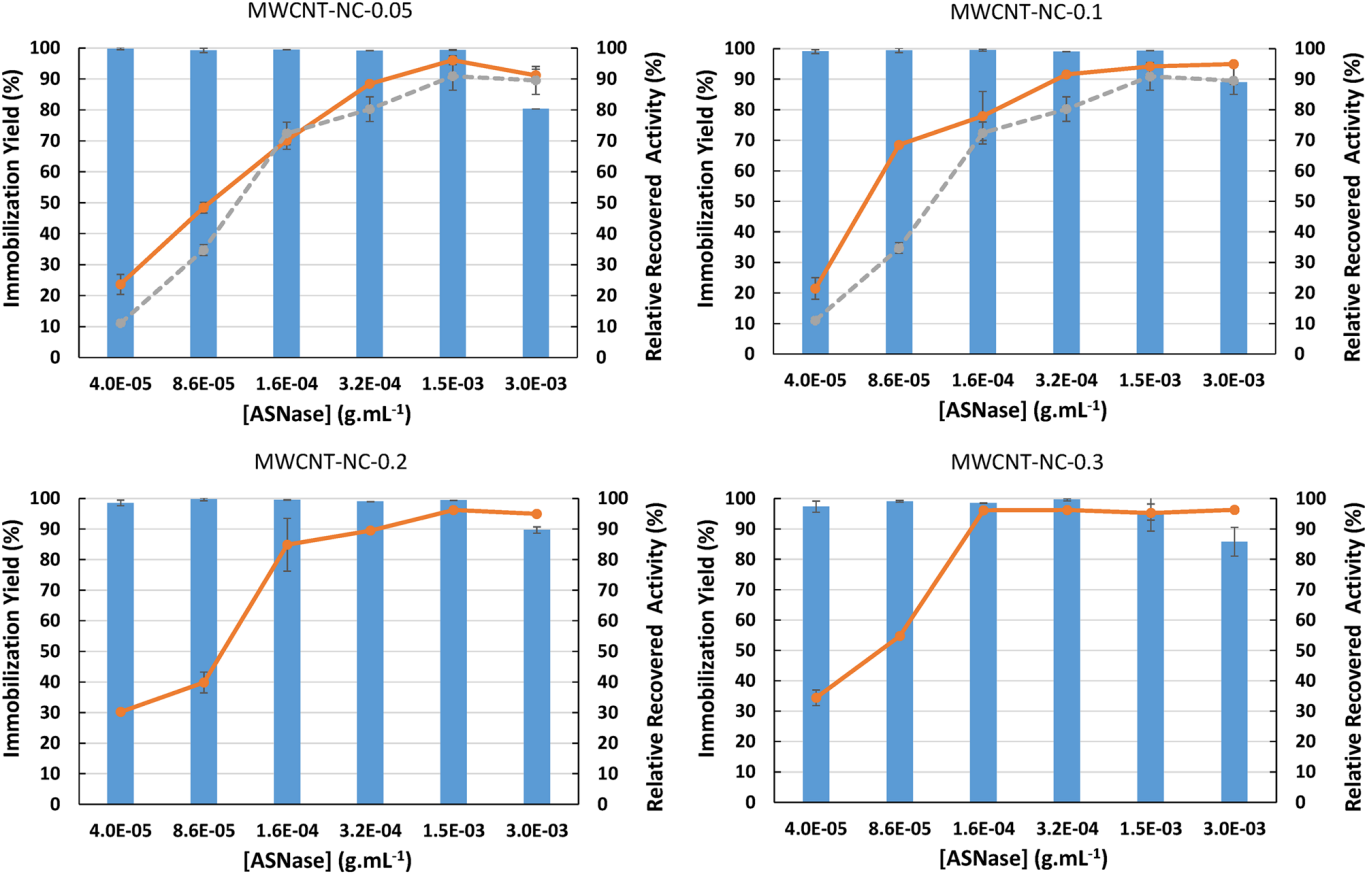
Effect of ASNase concentration on immobilization yield (columns) and relative recovered activity (orange lines) obtained with the immobilization of ASNase on 2 mg of functionalized MWCNTs (functionalization with HNO_3_ aqueous solutions with variable concentrations: 0.05, 0.10, 0.20 and 0.30 M) at pH 8 during 60 min. Grey lines represent the relative recovered activity of ASNase on 2 mg of MWCNTs without any functionalization (MWCNT-NC, control).

The ASNase concentration and materials adsorption capacity were finally investigated at the optimized pH and contact time (pH 8.0 and 60 min) for all MWCNTs. Six enzyme concentrations ranging between 4.0 × 10^–5^ g mL^−1^ (8.00 × 10^–6^ g of ASNase) and 3.0 × 10^–3^ g mL^−1^ (6.00 × 10^–4^ g of ASNase) were studied. The results obtained are presented in Fig. 3 (detailed data in Table S3 in the Supporting Information). Regarding the ASNase immobilization yield, it is possible to observe complete adsorption (100% of enzyme immobilization yield) for almost all the enzyme concentrations studied and for all the materials tested. Only when the highest concentration of enzyme studied was evaluated (3.0 × 10^–3^ g mL^−1^; 6.00 × 10^–4^ g of ASNase), a decrease in the immobilization yield was observed, but still higher than 80% (more than 2.40 × 10^–3^ g of ASNase was immobilized), suggesting that the maximum adsorption capacity was attained with a ASNase concentration of 1.5 × 10^–3^ g mL^−1^ (3.00 × 10^–4^ g of ASNase) for all the MWCNTs used. The ASNase RRA increases with ASNase concentration up to 1.5 × 10^–3^ g mL^−1^, corresponding to values higher than 90% of RRA. An exception is observed when using the MWCNT-NC 0.3, where the maximum RRA (higher than 95%) was achieved using an ASNase concentration of 1.6 × 10^–4^ g mL^−1^ (3.20 × 10^–5^ g of ASNase). This same behavior was observed for non-functionalized MWCNTs; however, the RRA values were lower when compared to modified MWCNTs, as explained above.

Similar ASNase immobilization yield (97.0%) was reported in the literature using calcium-alginate/MWCNTs hybrid beads^30^, although the immobilization approach used in our work may be considered more straightforward. On the other hand, Haroun et al.^31^ used a similar strategy to the one used in this work and applied oxidized MWCNTs to immobilize ASNase, but lower values of immobilization yield (54.4%) were achieved.

Adsorption isotherm studies were carried out to better understand the ASNase adsorption behavior over the MWCNTs. The experimental data were fitted to the Langmuir and Freundlich models (Eqs. 5 and 6, respectively). Taking into account the correlation coefficient (*R*^2^), the experimental equilibrium data were better fitted to the Langmuir model, with a *R*^2^ ranging between 0.90 and 0.94, while for the Freundlich model a *R*^2^ ranging between 0.72 and 0.80 was determined for the functionalized MWCNTs (Figure S2 and detailed data in Table S4 given in the Supporting Information). Similar results were described by Cristóvão et al.^29^ for non-functionalized MWCNTS. These results disclose the absence of ASNase multilayers formation on the support.

A good fit between the Langmuir model predictions and the experimental data (adsorption of several ASNase concentrations onto functionalized MWCNTs at pH 8 for 60 min) was observed (Fig. S2), indicating the formation of an ASNase monolayer on the support surface. The maximum ASNase adsorption capacity (*q*_*max*_) values are similar for all the functionalized MWCNTs studied: 152.5, 155.7, 156.3 and 157.6 U g^−1^ for MWCNT-NC-0.05, MWCNT-NC-0.1, MWCNT-NC-0.2 and MWCNT-NC-0.3, respectively. A slight increase in the adsorption capacity for all the functionalized nanomaterials was observed compared with the pristine MWCNTs (*q*_*max*_ for ASNase of 148.0 U g^−1^).

#### Operational stability of ASNase-MWCNTs bioconjugate

**Figure 4:**
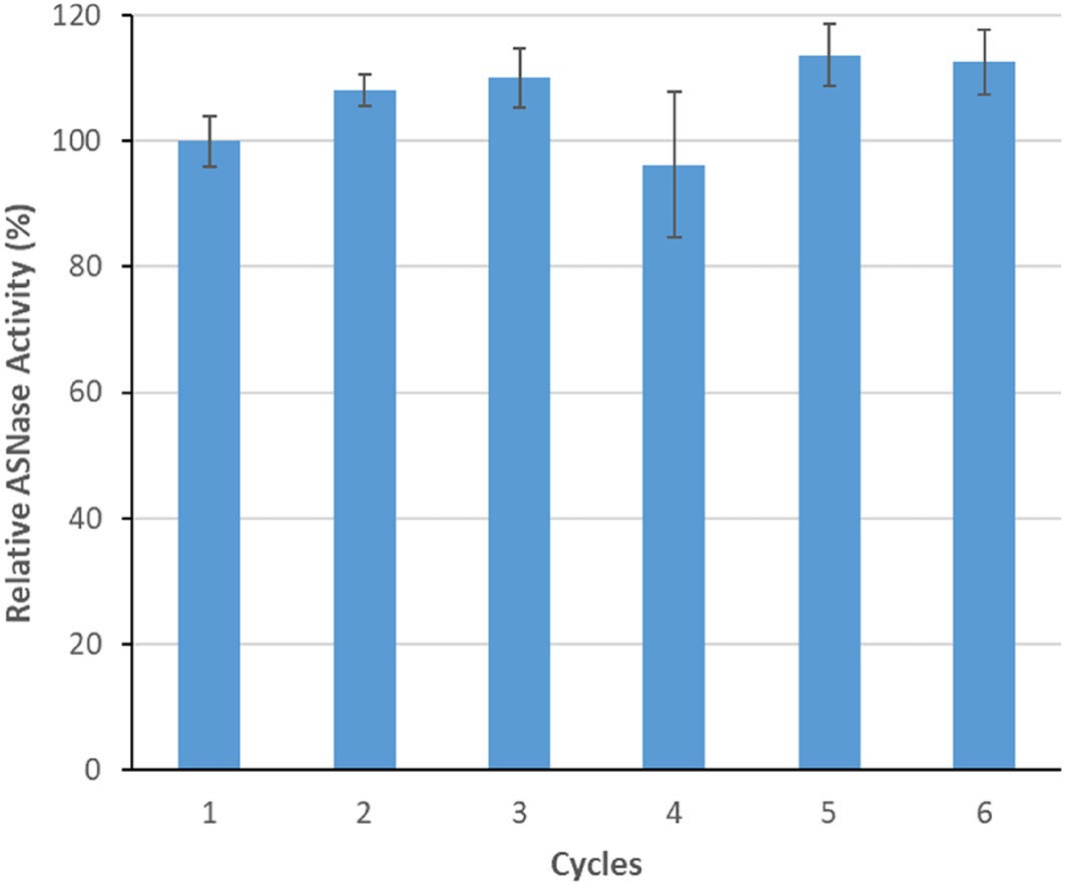
Operational stability of immobilized ASNase onto MWCNT-NC-0.3 during 60 min of contact time, pH 8.0, and with 1.5 × 10^–3^ g mL^−1^ of initial ASNase concentration.

Envisioning the industrial application of ASNase-MWCNTs in food or therapeutic sectors, the reusability of the bioconjugate is mandatory. This issue was studied by analyzing several cycles of l-asparagine hydrolysis using the most promising nanomaterial—MWCNT-NC-0.3 for the immobilization of 1.5 × 10^–3^ g mL^−1^ of ASNase. The results provided in Fig. 4 show that, despite using a simple ASNase adsorption method (where relatively weak interactions are expected to be involved), the overall system reveals exceptional operational stability, without any loss of immobilized ASNase activity during 6 consecutive reaction cycles. These results also emphasize the improved ASNase bioconjugate stability acquired after attachment onto functionalized MWCNTs. This confirmed stability is a relevant acquired advantage, allowing bioconjugate reuse several times in batch processes, as well as the application in continuous processing systems. Other authors have studied the immobilized ASNase operational stability during successive cycles using different supports; nevertheless, some activity losses have been reported. For example, Noma et al.^38^ reported 88% of ASNase initial activity after 5 reuse cycles when immobilized on Fe_3_O_4_/S_i_O_2_/NH_2_ and Fe_3_O_4_/COOH particles. In turn, Orhan and Uygun^39^ demonstrated that an ASNase-magnetic nanoparticles bioconjugate maintained 85.14% of its initial activity after 5 successive cycles. In the current work, with the ASNase-MWCNTs there are no losses on the immobilized enzyme activity for 6 cycles of reuse and reaction.

### ASNase-MWCNTs bioconjugate characterization

The texture and surface chemistry of pristine and functionalized MWCNTs were investigated to obtain an insight into the extent and nature of the interactions between the MWCNTs and ASNase (Table 1). This information is essential to better understand the improved enzyme immobilization yield and the respective RRA with the ASNase-MWCNTs bioconjugate.

**Table 1:** Surface area (S_BET_), concentrations of CO and CO_2_ obtained by TPD analysis and oxygen content obtained from elemental analysis.

	S_BET_ (m^2^ g^−1^)	[CO] (µmol g^−1^)	[CO_2_] (µmol g^−1^)	[CO] + [CO_2_]	[CO]/]CO_2_]	O (%)
MWCNT	189	95	25	120	3.80	0.25
MWCNT-NC-0.05	227	145	45	190	3.22	1.4
MWCNT-NC-0.1	262	903	291	1194	3.10	1.7
MWCNT-NC-0.2	271	1266	402	1668	3.15	2.4
MWCNT-NC-0.3	278	1541	489	2030	3.15	2.9

**Figure 5:**
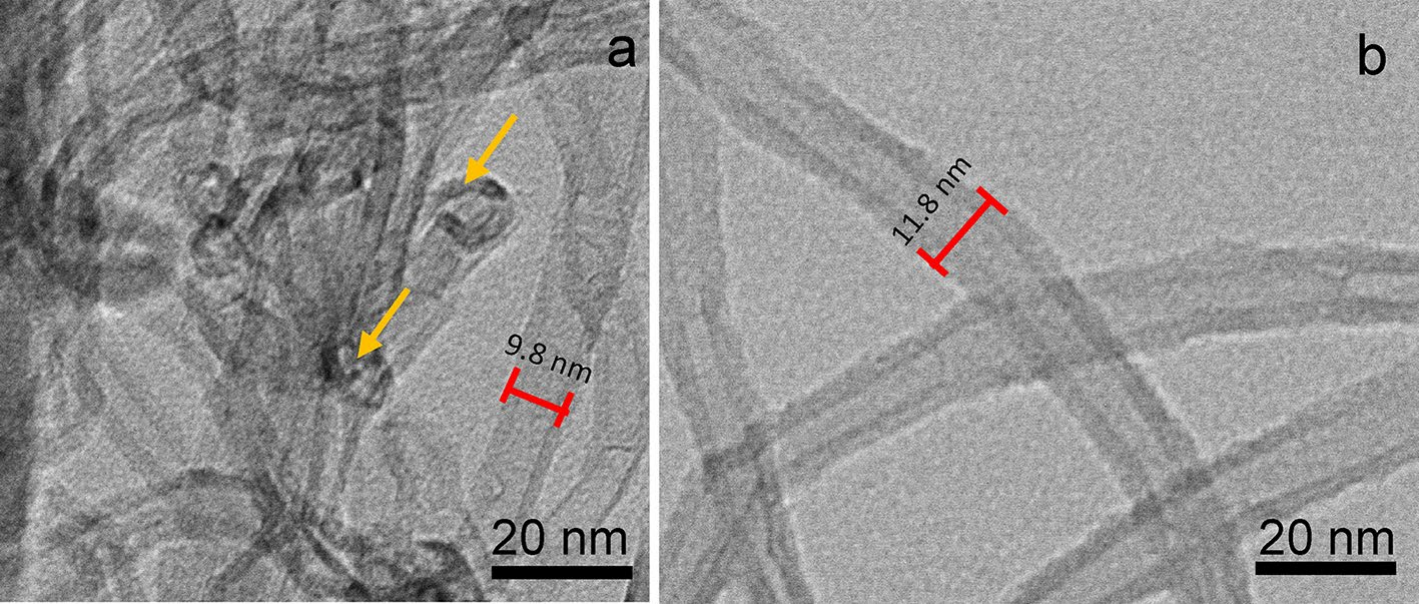
TEM micrographs of MWCNT-NC-0.3 (**a**) before and (**b**) after ASNase immobilization. The yellow arrows are marking the opened ends of the MWCNTs.

The acid treatment is known to introduce defects into the sidewalls of the MWCNTs as well as open up the end caps of the tubes (Fig. 5a)^40^, leading to an increase in the surface area (S_BET_) of the resulting materials. As the concentration of HNO_3_ used in the oxidation treatment increases, a progressive increase in the S_BET_ of the materials is observed (Table 1), which may be related to the improved ASNase adsorption ability of the MWCNTs. The S_BET_ varied from 189 m^2^ g^−1^ to 278 m^2^ g^−1^ for unmodified MWCNTs and MWCNT-NC-0.3, respectively.

The treatment with HNO_3_ produces materials with large quantities of surface oxygen groups, mainly carboxylic acids and, to a smaller extent, lactones, anhydrides, and phenol groups formed at the edges/ends and defects of graphitic sheets^41–43^. These groups are decomposed by heating the MWCNTs samples and are released in the form of CO and/or CO_2_, which were followed by temperature-programmed desorption (TPD) analysis (Figures S3 and S4 in Supporting Information). The total amounts of CO and CO_2_ released from the various CNT samples, obtained by integration of the TPD spectra, are presented in Table 1. The total amount of oxygen surface groups (released as CO and CO_2_) increase with the concentration of HNO_3_ used for the oxidation treatment, as can also be appraised by the percentage of oxygen determined by elemental analysis (Table 1). Moreover, the lower CO/CO_2_ ratio observed for the oxidized MWCNTs, compared with the pristine material, indicates a more acidic surface, which is mainly due to the presence of carboxylic acid groups.

The immobilization of 1.5 × 10^–3^ g mL^−1^ of ASNase over the MWCNTs was also confirmed by TEM, as displayed in Fig. 5 for MWCNT-NC-0.3 and ASNase-MWCNT-NC-0.3. Typical tubular morphology of MWCNTs was observed as well as the opened ends of the tubes (marked with arrows in Fig. 5a). After ASNase immobilization, the walls of the MWCNTs become thicker and more irregular due to the presence of the enzyme at the surface of the carbon nanomaterial (Fig. 5b). The difference between the thickness of the naked nanotubes and the ASNase-MWCNT-NC-0.3 bioconjugate gives an estimated thickness of the enzyme film of 2 nm.

**Figure 6:**
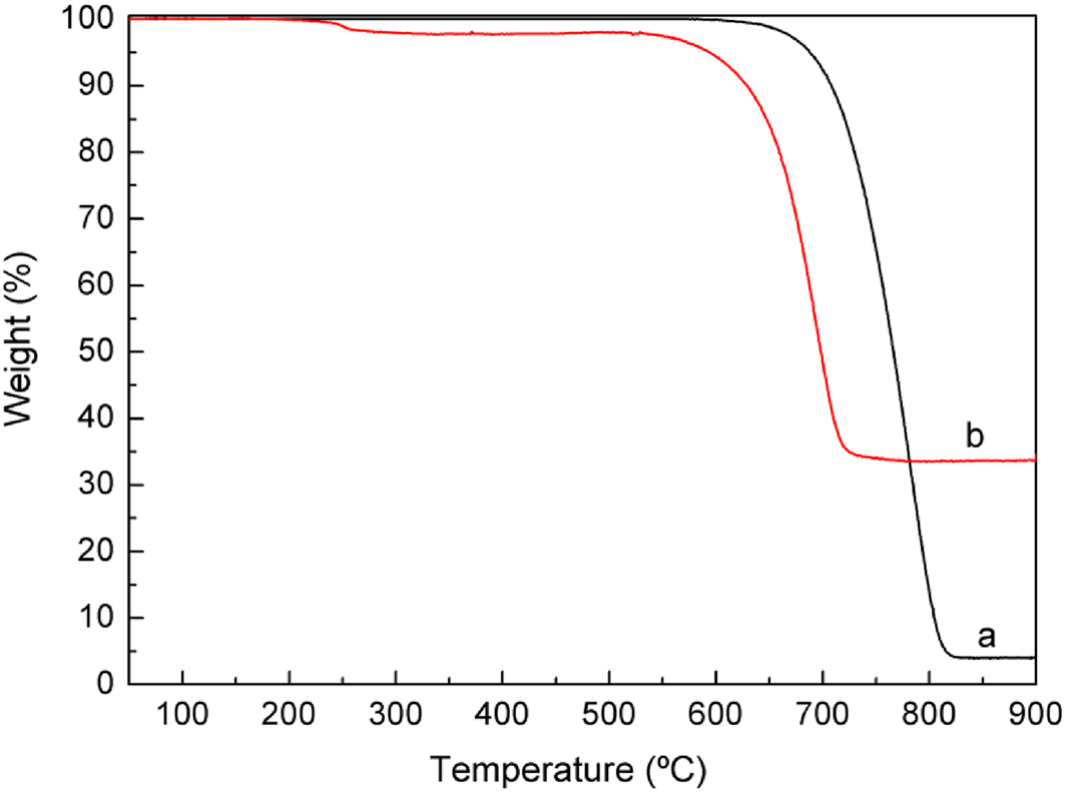
TGA analysis of MWCNT-NC-0.3 (**a**) before and (**b**) after ASNase immobilization.

Thermogravimetric analysis (TGA) of neat MWCNT-NC-0.3 and the respective ASNase bioconjugate, ASNase-MWCNT-NC-0.3 (immobilization with 1.5 × 10^–3^ g mL^−1^ of ASNase per 2 mg of material), is presented in Fig. 6. TGA results reveal that the MWCNT-NC-0.3 material starts to decompose at around 580 °C and is completely burned at 820 °C. At this temperature, a plateau is reached, corresponding to 3.9% of the initial mass, attributed to the presence of ashes resulting from the pyrolysis of inorganic impurities. Differently, two main weight losses are observed for the ASNase-MWCNT-NC-0.3 bioconjugate. The first weight loss is detected at c.a. 250 °C, which is attributed to the thermal decomposition of the enzyme^44^, while the second weight loss at 600 °C is recognized as the simultaneous pyrolysis of ASNase and MWCNTs, attaining a plateau at a temperature near to 700 °C. This plateau corresponds to 33.8% of the initial mass of the enzyme-support complex. These results suggest that the enzyme content of the bioconjugate should be c.a. 29.7 *wt.* % (difference between the weight loss obtained for the neat MWCNT-NC-0.3 and the bioconjugate), which corresponds to 0.422 g of enzyme *per* g of carbon nanotubes.

**Figure 7:**
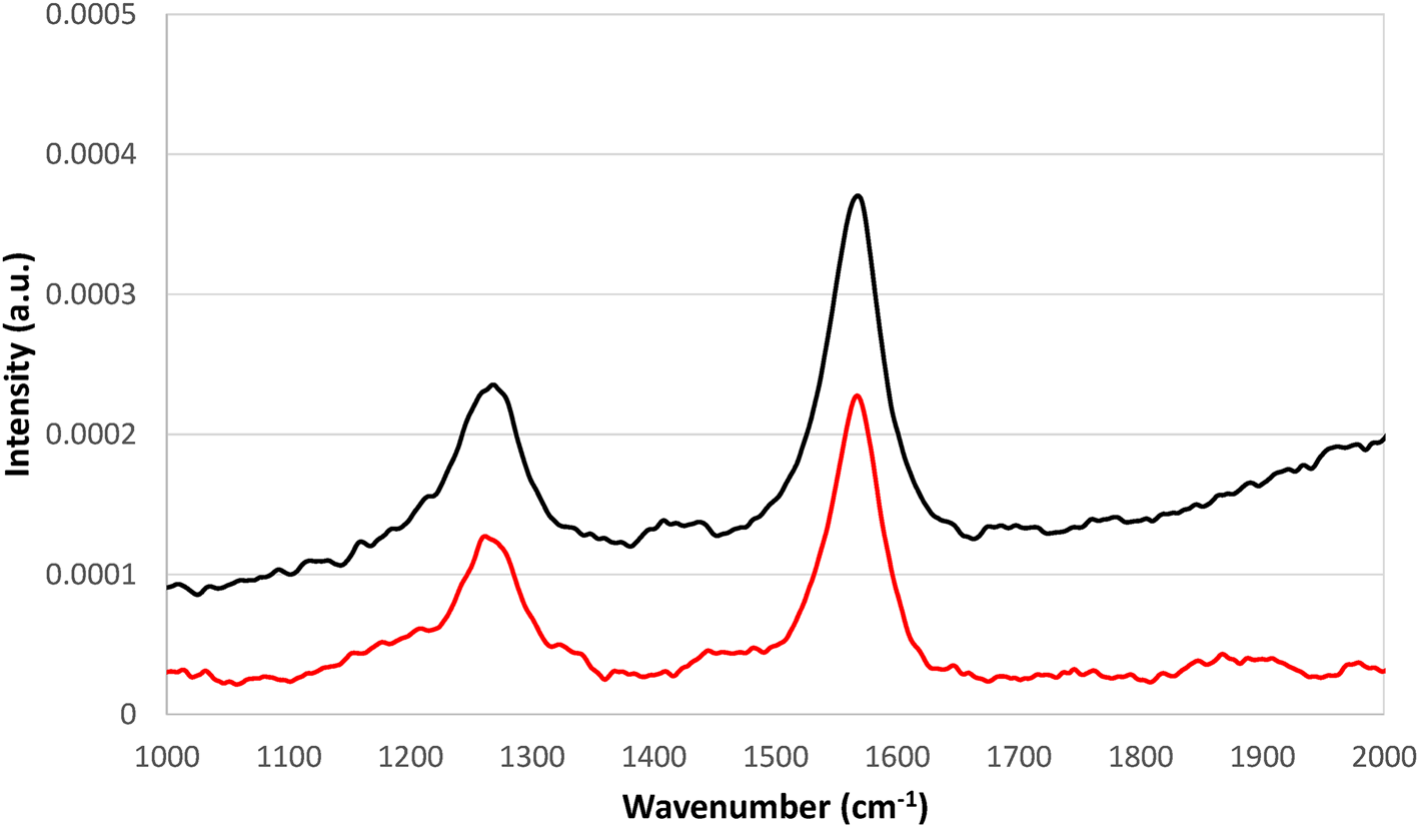
Raman spectra of MWCNT-NC-0.3 (black line) and ASNase-MWCNT-NC-0.3 bioconjugate (red line).

Raman spectroscopy was also used for characterizing the surface of the MWCNT-NC-0.3 before and after immobilization of 1.5 × 10^–3^ g mL^−1^ of ASNase. The Raman spectra exhibit two prominent characteristic bands in the spectral region between 1000 and 2000 cm^−1^ (Fig. 7). The G band, peaking at c.a. 1600 cm^−1^, corresponds to the MWCNTs regular sp^2^ graphitic network and is common to both materials, while the D band at 1287 cm^−1^ is assigned to the disorder and defects caused by sp^3^ hybridized carbon in the lattice^45^. The ratio between the intensities of D and G bands (I_D_/I_G_) gives information about the degree of disorder and the existence of defect sites in the MWCNTs lattice^46^. The I_D_/I_G_ values obtained for MWCNT-NC-0.3 and ASNase-MWCNT-NC-0.3 were 0.636 and 0.631, respectively. These similar values suggest that the enzyme immobilization in the nanomaterials do not cause a significant disturbance on the CNTs surface, indicating similar degrees of disorder, i.e., ASNase adsorption occurs predominantly on the defect sites present at the MWCNT-NC-0.3 surface, not changing the sp^2^ and sp^3^ bonds concentration.

## Conclusions

The hydrothermal functionalization of MWCNTs significantly improves the interaction with the ASNase enzyme. The MWCNTs functionalized with 0.3 M HNO_3_ aqueous solution produced the highest immobilization yield and relative recovered activity of ASNase above 95%. With these levels of immobilization and activity, one can unlock higher stages of application for this ASNase-MWCNT bioconjugate, like new drug carriers development for concrete targets (e.g., aggressive cancer types) and a wider application in the food industry.

Tunning of the process could be done by controlling the pH value, contact time between the enzyme and the support, and ASNase/MWCNTs mass ratio during the immobilization procedure. The ASNase/MWCNTs mass ratio was the parameter with the most substantial effect on improving immobilization.

The optimized adsorption conditions (using the Langmuir model) to achieve an immobilization yield and a relative recovery activity of ASNase above 95% consisted of pH 8.0, 60 min of contact time and enzyme concentration of 1.5 × 10^–3^ g mL^−1^ in 2.0 mg of MWCNTs. These conditions correspond to an MWCNT maximum predicted enzyme loading capacity of 158.0 U g^−1^. Because the adsorption results followed well a Langmuir isotherm behavior, it can be assumed that the adsorption of an ASNase on the support consists of a monolayer. It results in excellent operational stability, with the ASNase immobilization onto MWCNTs without any ASNase activity loss for 6 reaction cycles. Enzymatic activity and stability, which are dependent on the oxidized MWCNT surfaces, types of interactions formed and concentrations used, resulted in exceptional operational stabilities under optimized conditions. This demonstrates the potential for improved industrial applications, where the prepared catalyst can be easily separated and reused several times, reducing the demand for the enzyme and improving the economy of the process. All of these advantages outweigh the technical requirements needed to modify the MWCNTs.

In general, the provided straightforward approach demonstrates how functionalization of MWCNTs can be tailored to support ASNase, or any other enzyme efficiently, opening the possibility to produce specific bioconjugates for several application fields, e.g., as biosensors and the pharmaceutical and food industry.

## Experimental section

### Materials

Lyophilized ASNase (with no additives; purity > 96.0%) from *E. coli* ASI.357 (ENZ-287) was supplied by Prospec. Multi-walled carbon nanotubes (MWCNTs) with average diameter and length of 9.5 nm and 1.5 μm, respectively, specific surface area (S_BET_) of 189 m^2^ g^−1^ and purity ≥ 95% were purchased from Nanocyl (NC3100). Nitric acid used for MWCNTs functionalization was supplied by Sigma-Aldrich. l-asparagine (≥ 99.0%) was supplied by Acros Organics. Disodium phosphate heptahydrate (98.0–102%), sodium carbonate (99.0%), and sodium hydrogen carbonate (99.5%) were acquired from Sigma-Aldrich, Vencilab and Prolabo, respectively. Tris(hydroxymethyl)aminomethane (TRIS) (≥ 99.8%), trichloroacetic acid (TCA) (Analytic), and Nessler’s reagent were purchased from Prolabo, Prunella and Fluka, respectively.

### MWCNTs functionalization

Traditional methods for the introduction of oxygen functionalities at the surface of carbon materials include hydrothermal oxidation with nitric and sulphuric acids, and with hydrogen peroxide, and also thermal treatment with oxygen in gas phase. In previous works on the immobilization of other enzymes, such as laccase and peroxidase over MWCNTs, we found that the presence of hydroxyl groups are beneficial for improving the interaction of the proteins with the surface of the carbon nanotubes^21,22^. In those works, the functionalization consisted in the oxidation of the MWCNTs by heating at reflux in a highly concentrated HNO_3_ aqueous solution (7 M), followed by washing with distilled water until neutral pH. In fact, that process generated high volumes of residual rinsing water containing nitric acid. Therefore, in the present work, we used an alternative approach consisting of a high-pressure hydrothermal treatment, which allowed the use of much less concentrated nitric acid solutions (0.1 – 0.3 M) and half the volume of solution to treat the same amount of MWCNTs^12,47^. Moreover, the amount of oxygen functionalities introduced at the surface of the carbon nanomaterials was similar using both techniques.

In this work, hydrothermal oxidation of the pristine MWCNTs was carried out in a Teflon-lined stainless-steel autoclave using HNO_3_ aqueous solutions with variable concentrations (0.05, 0.10, 0.20 and 0.30 M) at 200 °C, as described elsewhere^12^. Briefly, 0.2 g of MWCNTs was added to 75 mL of an HNO_3_ aqueous solution. After being sealed, the vessel was put into an oven at 200 °C for 2 h. Then, the MWCNTs were recovered, rinsed with water until neutrality, and dried overnight at 120 °C. The resulting materials are labeled as MWCNT-NC-Y, where Y corresponds to the HNO_3_ concentration (in M) used in the oxidation treatment (Y = 0.05, 0.10, 0.20 and 0.30 M). These materials were used for the subsequent ASNase immobilization.

### ASNase immobilization on functionalized MWCNTs

The ASNase immobilization over the functionalized MWCNTs was carried out by direct physical adsorption in batch system. Several parameters were studied to maximize the ASNase adsorption, namely pH, contact time and ASNase/MWCNT mass ratio, and reported in terms of immobilization yield and relative recovered ASNase activity. In a typical experiment, MWCNTs (2.0 ± 0.1 mg) were added to 200 μL of an ASNase solution, and the suspensions were left in contact in an orbital stirrer, as detailed below.

The pH effect on the ASNase adsorption and relative recovered activity were studied using a 0.15 M citrate–phosphate buffer at pH value 5.0 and a 0.2 M phosphate buffer at pH values of 6.0, 7.0 and 8.0. For that purpose, 200 μL of ASNase (final concentration of 8.6 × 10^–5^ g mL^−1^; 1.72 × 10^–5^ g of ASNase) was prepared in each studied buffer and added to the MWCNTs. This mixture was stirred for 60 min in an orbital shaker at 50 rpm. A control was also prepared using free ASNase at 8.6 × 10^–5^ g mL^−1^ (1.72 × 10^–5^ g of ASNase) in each of the evaluated buffers.

The influence of the contact time between the ASNase and the MWCNTs was also investigated. The experiments were performed by adding 200 μL of ASNase 8.6 × 10^–5^ g mL^−1^ (1.72 × 10^–5^ g of ASNase) in 0.2 M phosphate buffer at pH 8.0 to the MWCNTs. The pH 8.0 was selected, taking into account the results obtained in the previous assays. The mixture was stirred during periods ranging between 15 and 120 min in an orbital shaker at 50 rpm. A control sample was prepared using 200 μL of ASNase 8.6 × 10^–5^ g mL^−1^ (1.72 × 10^–5^ g of ASNase) in phosphate buffer at pH 8.0.

Finally, the effect of ASNase concentration was evaluated by adding 200 μL of different ASNase concentrations, from 4.0 × 10^–5^ (8.00 × 10^–6^ g of ASNase) to 3.0 × 10^–3^ (6.00 × 10^–4^ g of ASNase) g mL^−1^, at pH 8.0, to the MWCNTs. The mixture was stirred for 60 min in an orbital shaker. Both pH value and contact time were selected, taking into account the results obtained in the previous assays. A control was prepared with 200 μL of ASNase in phosphate buffer at pH 8.0 at each concentration.

At least three individual experiments were carried out for each condition, allowing the determination of the average immobilization yield and relative recovered activity and respective standard deviations.

### ASNase activity determination

The enzyme activity was determined by quantifying ammonia released after the l-asparagine (substrate) hydrolysis by ASNase. The experimental procedure comprises the mixture of 50 µL of l-asparagine (189 mM) with 50 µL of enzyme solution (initial free enzyme or supernatant after immobilization) or with 2.0 mg of attached ASNase on MWCNTS (immobilized enzyme) in 500 µL of TRIS–HCl buffer (50 mM and pH 8.6), and 450 µL of deionized water, at 37 °C for 30 min under stirring. After incubation, for the free enzyme, the reaction was stopped by adding 250 µL of 1.5 M TCA, while for the immobilized enzyme, the supernatant (containing ammonia) was separated throughout centrifugation at 12 000 rpm during 15 min. Subsequently, 100 µL of the previous free enzyme solution or 100 µL of the supernatant was mixed with 2.15 mL of deionized water and 250 µL of Nessler’s reagent to measure the ammonia amount^48^. After 30 min of incubation, the increase in absorbance was measured by spectroscopy, using a BioTeck Synergy HT microplate reader at 436 nm. A calibration curve was previously established using ammonium sulfate. Possible interferences of CNTs with Nessler’s reagent were investigated, and no interference was found.

One unit of free ASNase activity is defined as the amount of enzyme that releases 1 µmol of ammonia per minute at 37 °C (Eq. 1):

1 $$ASNase\,activity \left(\frac{U}{L}\right)= \frac{\left[{NH}_{4}^{+}\right]\left(\frac{\mu mol}{mL}\right)\times {V}_{Nessler}\left(mL\right)\times {f}_{d}}{{t}_{r}(\text{min})}$$

where *V*_*Nessler*_ is the volume of the Nessler solution, *f*_*d*_ is the sample dilution factor, and *t*_*r*_ is the reaction time.

One unit of immobilized ASNase activity is defined as the amount of enzyme that releases 1 µmol of ammonia *per* minute and mass of support at 37 °C (Eq. 2).

2 $$ASNase\,activity \left(\frac{U}{mg}\right)= \frac{[{NH}_{4}^{+}]\left(\frac{\mu mol}{mL}\right)\times {V}_{Nessler}\left(mL\right)\times {f}_{d}}{{t}_{r}(\text{min})\times {m}_{s}(mg)}$$

where *m*_*s*_ is the mass of the support.

The immobilization yield, *IY* (%), is defined as the difference between the free enzyme activity before immobilization and the activity of the free enzyme remaining in the supernatant after immobilization divided by the free enzyme activity before immobilization (Eq. 3).

3 $$IY \left(\%\right)=\frac{Free\,ASNase\,Activity\left(\frac{U}{mL}\right)-Supernatant\,ASNase\,Activity \left(\frac{U}{mL}\right)}{Free\,ASNase\,Activity \left(\frac{U}{mL}\right)}\times 100$$

The relative recovered activity, *RRA* (%), of the immobilized enzyme, was calculated as the ratio between the activity of the effectively immobilized enzyme and the maximum theoretical activity that would exist if the free enzyme was totally immobilized (Eq. 4).

4 $$RRA \left(\%\right)=\frac{Immobilized\,ASNase\,activity\left(\frac{U}{mg}\right)}{Maximum\,ASNase\,activity\left(\frac{U}{mg}\right)}$$

where: $$Maximum\,ASNase\,activity \left(\frac{U}{mg}\right)=\frac{{[{NH}_{4}^{+}]}_{free ASNase}\left(\frac{\mu mol}{mL}\right)\times {V}_{Nessler}\left(mL\right)\times {f}_{d}}{{t}_{r}(\text{min})\times {m}_{s}(mg)}$$

### Adsorption isotherms

The adsorption equilibrium behavior of the ASNase onto MWCNTs was evaluated by adjusting the experimental results to the Langmuir and Freundlich isotherm models. The ASNase concentrations were varied from 4.0 × 10^–5^ (8.00 × 10^–6^ g of ASNase) to 3.0 × 10^–3^ (6.00 × 10^–4^ g of ASNase) g mL^−1^ at pH 8.0, and the equilibrium time was fixed at 60 min.

The Langmuir model parameters were determined by fitting Eq. 5 to the experimental data using the CurveExpert v 1.38 software:

5 $$q=\frac{{q}_{max}\times K\times C}{1+K\times C}$$

where *q* is the amount of adsorbed active ASNase (U) *per* gram of MWCNT (U g^−1^), *q*_*max*_ is the maximum adsorption capacity of active ASNase (U g^−1^), *K* is the Langmuir adsorption equilibrium constant (mL g^−1^) related to the strength of affinity between the protein and the surface^49^ and *C* is the ASNase concentration (g mL^−1^).

The Freundlich model parameters were estimated by fitting of Eq. (6) to the experimental data using the CurveExpert v 1.38 software:

6 $$q = k_{F} \times C^{{{1 \mathord{\left/ {\vphantom {1 n}} \right. \kern-\nulldelimiterspace} n}}}$$

where *k*_*F*_ is the Freundlich binding constant related to the adsorption of ASNase *per* weight of MWCNT [(U g^−1^)·(mL g^−1^)^(1/n)^], and *n* is an empirical parameter that is a measure of the intensity of adsorption in Freundlich adsorption isotherms.

### Operational stability of immobilized ASNase

The operational stability of ASNase-MWCNTs bioconjugate was assessed by incubating 2 mg of the immobilized ASNase with 50 µL of l-asparagine (189 mM) in 500 µL of TRIS–HCl buffer (50 mM and pH 8.6), and 450 µL of deionized water, at 37 °C for 30 min under stirring. At the end of each cycle, the reaction was stopped by the supernatant removal throughout centrifugation at 12 000 rpm for 15 min, and subsequent addition of 250 µL of TCA 1.5 M in the supernatant. The immobilized ASNase was washed twice with phosphate buffer pH 8.0 (± 500 µL each wash) and resuspended in a fresh substrate solution to begin the next cycle. Six cycles of operational stability were carried out, and for each assay triplicate runs were performed.

### Determination of the isoelectric point of ASNase

The isoelectric point (IP) of ASNase was determined by the measurements of zeta potential of aqueous solutions of ASNase in a wide pH range, between 3 and 11 (pH adjusted with HCl and NaOH solutions), using a Malvern Zetasizer Nano ZS (Malvern Instruments Ltd. Malvern) instrument at room temperature (25 °C). Aqueous solutions of NaOH or HCl 0.01 M were used to adjust the pH of the ASNase solutions.

### MWCNTs and ASNase-MWCNTs bioconjugate characterization

The textural properties of the MWCNT materials were determined by N_2_ adsorption–desorption at − 196 °C in the relative pressure range 0.05–0.15 on a Quantachrome NOVA 4200e apparatus. Before analysis, each sample (c.a. 100 mg) was degassed in vacuum at 120 °C for 3 h. The specific surface area (*S*_*BET*_) was calculated by multipoint BET analysis of the obtained data.

Temperature programmed desorption (TPD) analysis was carried out by heating the samples to 1100 °C at 5 °C min^−1^ under helium flow using an AMI-300 Catalyst Characterization Instrument (Altamira Instruments). The total amounts of CO and CO_2_ evolved from the samples were obtained by integration of the TPD spectra. The oxygen content was determined using a rapid oxy cube analyzer (Elementar GmbH), in which the sample underwent pyrolysis at 1450 °C. Each sample was analyzed in triplicate. Thermogravimetric (TG) analysis studies were performed on an STA 490 PC/4/H Luxx Netzsch thermal instrument. For each analysis, samples with approximately 10 mg were loaded on an alumina crucible and heated at 10 °C min^−1^ from 50 °C to 900 °C under airflow, while the weight was measured and recorded continuously.

Raman spectra were recorded in a Brucker RFS100/S FT-Raman spectrometer (Nd:YAG laser, 1064 nm excitation), at a power of 200 mV, with 3000 scans at a resolution of 4 cm^−1^.

A JEOL 2010F analytical electron microscope, equipped with a field-emission gun, was used for transmission electron microscopy (TEM) images.

## Supplementary Information

Supplementary Information.
