# Supplementary material for: Superior operational stability of immobilized l-asparaginase over surface-modified carbon nanotubes
Source: Sci Rep. 2021 Nov 2;11:21529. doi: 10.1038/s41598-021-00841-2 (PMC8563809; doi:10.1038/s41598-021-00841-2)
Supplement: Supplementary file 1 — Supplementary Information. [file 41598_2021_841_MOESM1_ESM.pdf]

Superior operational stability of immobilized L-asparaginase over surface-modified carbon nanotubes

Mafalda R. Almeida[1†], Raquel O. Cristóvão[2†], Maria A. Barros[2], João C. F. Nunes[1], Rui A. R. Boaventura[2], José M. Loureiro[2], Joaquim L. Faria[2], Márcia C. Neves[1], Mara G. Freire[1], Valéria C. Santos-Ebinuma[3], Ana P. M. Tavares[1*], Cláudia G. Silva[2*]

[1]*CICECO-Aveiro Institute of Materials, Department of Chemistry, University of Aveiro, 3810-193 Aveiro, Portugal*

[2]*Laboratory of Separation and Reaction Engineering - Laboratory of Catalysis and Materials (LSRE-LCM), Department of Chemical Engineering, Faculty of Engineering, University of Porto, Rua do Dr. Roberto Frias, 4200-465, Porto, Portugal*

[3]*Department of Engineering Bioprocess and Biotechnology, School of Pharmaceutical Sciences, UNESP-University Estadual Paulista, Araraquara, Brazil*

[†]These authors contributed equally to this work.

*Corresponding authors:

Cláudia G. Silva:
Tel: +351 220 414 874; *e-mail:* cgsilva@fe.up.pt

Ana P. M. Tavares:
Tel: +351 234 401 520; *e-mail*: aptavares@ua.pt

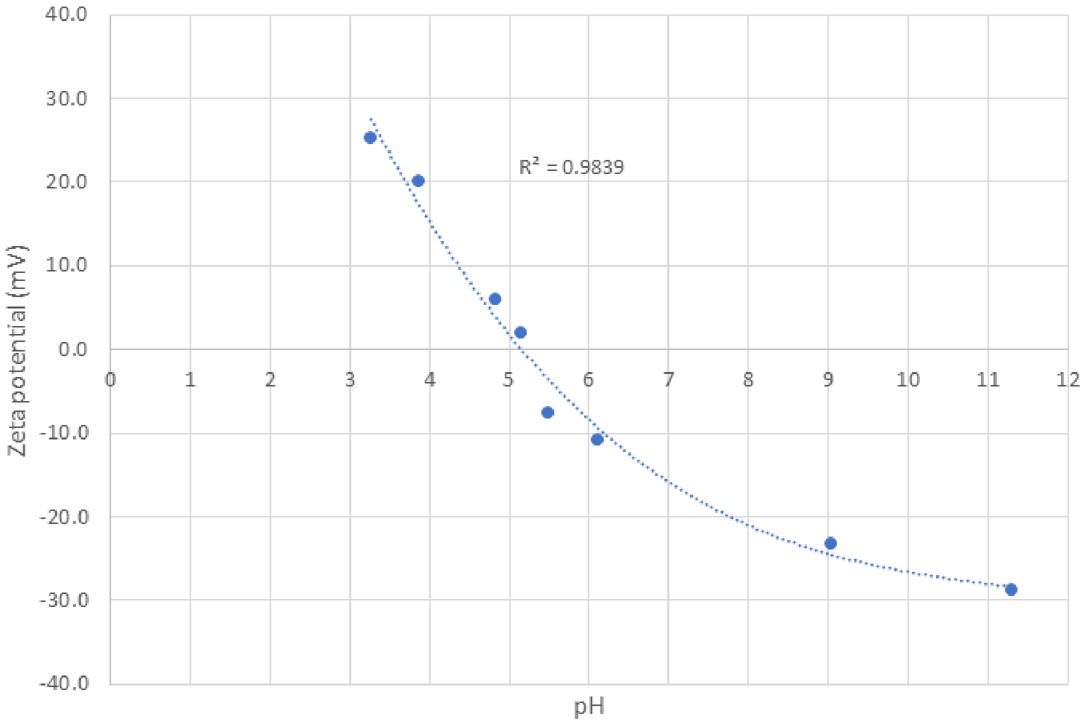

Figure S1. Zeta potential of ASNase 8.6×10^{-5} g.mL^{-1} at different pH values.

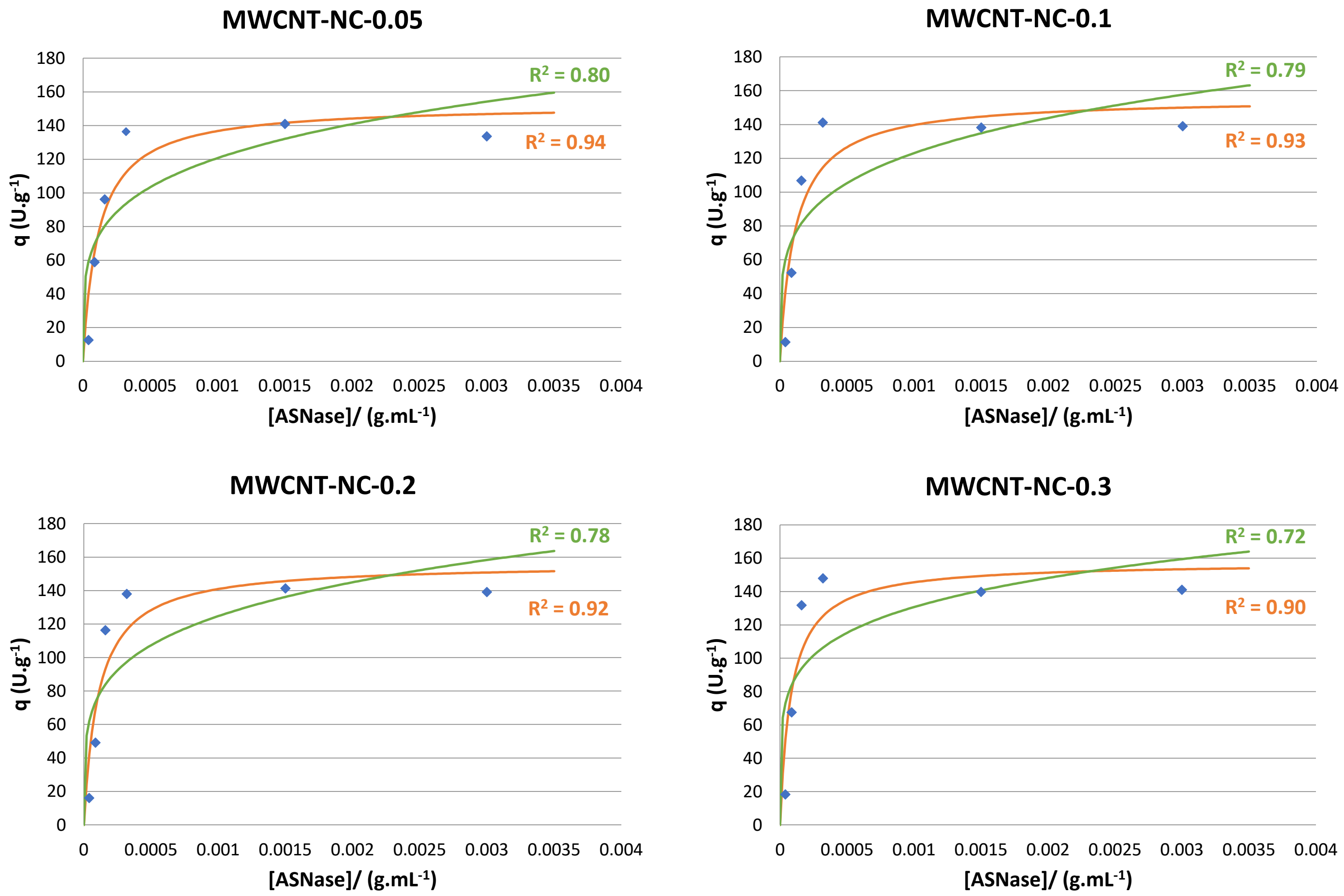

Figure S2. Langmuir (orange line) and Freundlich (green line) isotherm model predictions and experimental data (dots) for the immobilization of different concentrations of ASNase on 2 mg of functionalized MWCNTs (functionalization with HNO_3 aqueous solutions with variable concentrations: 0.05, 0.10, 0.20 and 0.30 M) at pH 8 during 60 min.

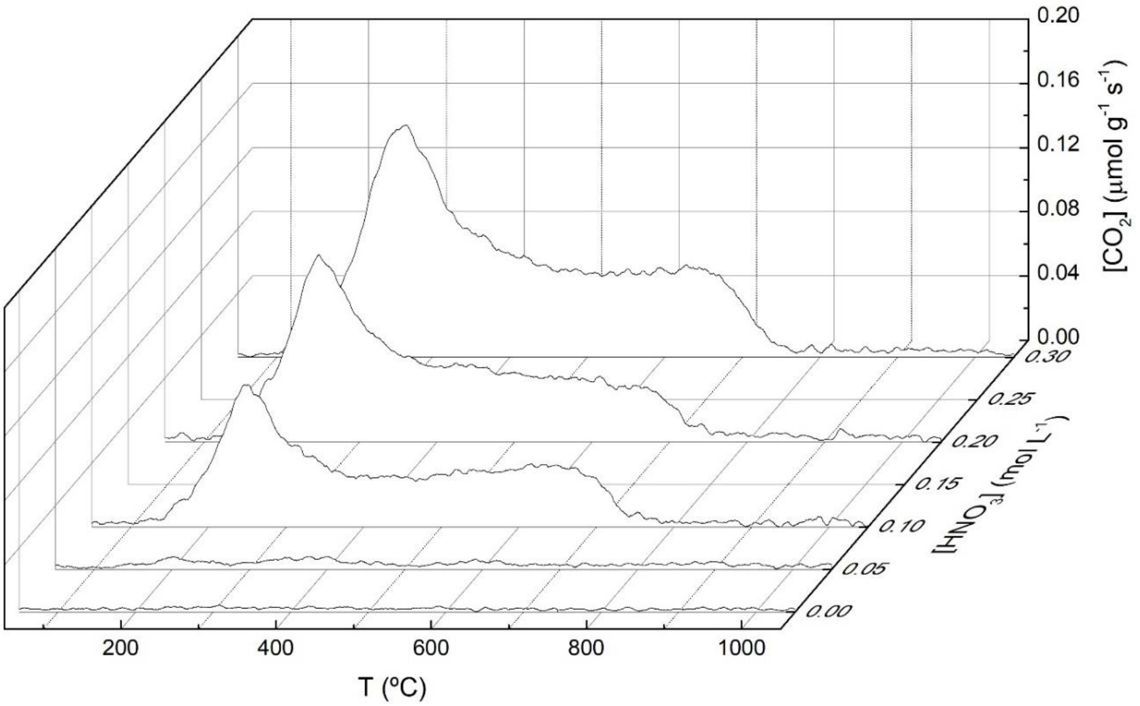

Figure S3. Temperature-programmed desorption (TPD) spectra along with the amount of CO_2 released from the various CNT samples *per* second

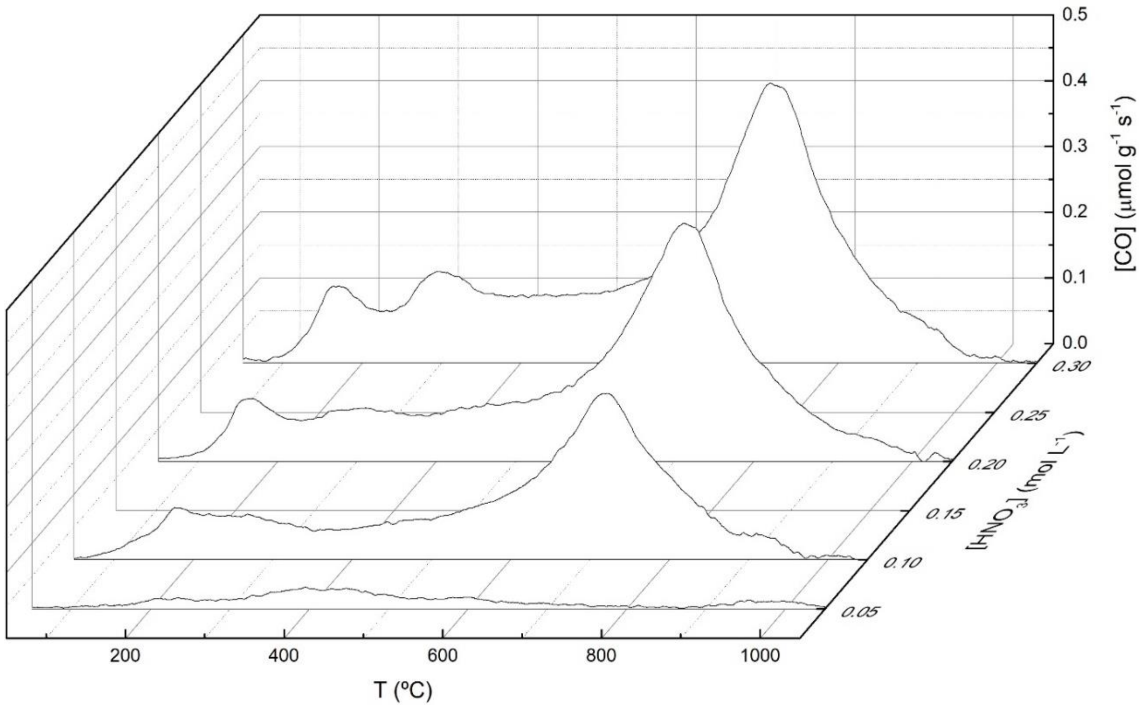

Figure S4. Temperature-programmed desorption (TPD) spectra with the amount of CO released from the various CNT samples *per* second

Table S1. Effect of pH on immobilization yield and relative recovered activity (RRA) obtained with the immobilization of 8.6×10^{-5} $g.mL^{-1}$ of ASNase on 2 mg of MWCNTs after 60 min of contact time.

pH	RRA (%)				Immobilization yield (%)			
	CNT-NC 0.05	CNT-NC 0.1	CNT-NC 0.2	CNT-NC 0.3	CNT-NC 0.05	CNT-NC 0.1	CNT-NC 0.2	CNT-NC 0.3
5	23 ± 1	26 ± 1	20 ± 1	34 ± 1	100 ± 2	100 ± 2	100 ± 2	100 ± 2
6	34 ± 2	37 ± 2	34 ± 2	35 ± 1	100 ± 2	100 ± 2	99 ± 2	99 ± 2
7	33 ± 2	36 ± 2	43 ± 2	40 ± 1	100 ± 2	100 ± 1	100 ± 2	100 ± 2
8	48 ± 2	69 ± 3	65 ± 3	57 ± 3	100 ± 2	100 ± 1	100 ± 1	100 ± 2

Table S2. Effect of contact time on immobilization yield and relative recovered activity (RRA) obtained with the immobilization of 8.6×10^{-5} $g.mL^{-1}$ of ASNase on 2 mg of MWCNTs after 60 min of contact time.

Time (min)	RRA (%)				Immobilization yield (%)			
	CNT-NC 0.05	CNT-NC 0.1	CNT-NC 0.2	CNT-NC 0.3	CNT-NC 0.05	CNT-NC 0.1	CNT-NC 0.2	CNT-NC 0.3
15	33 ± 2	29 ± 2	37 ± 2	13 ± 1	75 ± 1	73 ± 2	71 ± 1	68 ± 1
45	48.± 2	54 ± 3	47 ± 2	22 ± 1	96 ± 2	95 ± 2	98 ± 2	98 ± 2
60	56 ± 2	69 ± 3	65 ± 3	57 ± 3	100 ± 1	100 ± 2	100 ± 2	100 ± 2
90	56 ± 3	63 ± 3	64 ± 3	58 ± 3	94 ± 2	97 ± 2	100 ± 2	96 ± 2
120	55 ± 3	66 ± 3	65 ± 3	56 ± 2	100 ± 1	100 ± 2	100 ± 1	100 ± 1

Table S3. Effect of enzyme concentration on immobilization yield and relative recovered activity (RRA) obtained with the immobilization of 8.6×10^{-5} g.mL^{-1} of ASNase on 2 mg of MWCNTs after 60 min of contact time.

ASNase concentration ($\times10^{-3}$ g.mL^{-1})	RRA (%)				Immobilization yield (%)			
	CNT-NC 0.05	CNT-NC 0.1	CNT-NC 0.2	CNT-NC 0.3	CNT-NC 0.05	CNT-NC 0.1	CNT-NC 0.2	CNT-NC 0.3
0.040	24 ± 3	22 ± 4	30 ± 1	34 ± 3	99.8 ± 0.3	99.0 ± 0.6	99 ± 1	97 ± 2
0.086	48 ± 2	68.5 ± 0.6	65 ± 3	57 ± 1	99.2 ± 0.6	99.5 ± 0.7	99.7 ± 0.4	99.1 ± 0.3
0.160	70 ± 3	78 ± 8	85 ± 9	96.1 ± 0.2	99.5 ± 0.1	99.5 ± 0.3	99.5 ± 0.5	98.5 ± 0.1
0.320	88.4 ± 0.2	91.6 ± 0.1	89.5 ± 0.1	96 ± 1	99.1 ± 0.1	99.0 ± 0.1	99.0 ± 0.1	99.6 ± 0.4
1.500	96 ± 1	94 ± 1	96 ± 1	95 ± 6	99.3 ± 0.1	99.4 ± 0.1	99.4 ± 0.1	96 ± 3
3.000	91 ± 2	95 ± 1	95 ± 1	96.3 ± 0.2	80.3 ± 0.1	89 ± 1	90 ± 1	86 ± 5

Table S4. Amount of adsorbed active ASNase (U) per gram of functionalized MWCNT ($U.g^{-1}$) for immobilizing different concentrations of ASNase on 2 mg of functionalized MWCNTs with different HNO_3 concentration, at pH 8 during 60 min, used for the prediction of Langmuir and Freundlich isotherm models.

ASNase concentration ($\times 10^{-3}$ $g\cdot mL^{-1}$)	CNT-NC 0.05	CNT-NC 0.1	CNT-NC 0.2	CNT-NC 0.3
	q ($U\cdot g^{-1}$)	q ($U\cdot g^{-1}$)	q ($U\cdot g^{-1}$)	q ($U\cdot g^{-1}$)
0.040	12.57	11.42	16.06	18.30
0.086	58.86	52.40	49.11	67.47
0.160	96.19	106.85	116.34	131.78
0.320	136.38	141.24	138.00	147.91
1.500	140.97	138.21	141.21	139.76
3.000	133.58	139.10	139.10	141.07
